# Facing Aggression: Cues Differ for Female versus Male Faces

**DOI:** 10.1371/journal.pone.0030366

**Published:** 2012-01-20

**Authors:** Shawn N. Geniole, Amanda E. Keyes, Catherine J. Mondloch, Justin M. Carré, Cheryl M. McCormick

**Affiliations:** 1 Department of Psychology, Brock University, St. Catharines, Ontario, Canada; 2 Centre for Neuroscience, Brock University, St. Catharines, Ontario, Canada; 3 Department of Psychology, Wayne State University, Detroit, Michigan, United States of America; Tel Aviv University, Israel

## Abstract

The facial width-to-height ratio (face ratio), is a sexually dimorphic metric associated with actual aggression in men and with observers' judgements of aggression in male faces. Here, we sought to determine if observers' judgements of aggression were associated with the face ratio in female faces. In three studies, participants rated photographs of female and male faces on aggression, femininity, masculinity, attractiveness, and nurturing. In Studies 1 and 2, for female and male faces, judgements of aggression were associated with the face ratio even when other cues in the face related to masculinity were controlled statistically. Nevertheless, correlations between the face ratio and judgements of aggression were smaller for female than for male faces (F_1,36_ = 7.43, *p* = 0.01). In Study 1, there was no significant relationship between judgements of femininity and of aggression in female faces. In Study 2, the association between judgements of masculinity and aggression was weaker in female faces than for male faces in Study 1. The weaker association in female faces may be because aggression and masculinity are stereotypically male traits. Thus, in Study 3, observers rated faces on nurturing (a stereotypically female trait) and on femininity. Judgements of nurturing were associated with femininity (positively) and masculinity (negatively) ratings in both female and male faces. In summary, the perception of aggression differs in female versus male faces. The sex difference was not simply because aggression is a gendered construct; the relationships between masculinity/femininity and nurturing were similar for male and female faces even though nurturing is also a gendered construct. Masculinity and femininity ratings are not associated with aggression ratings nor with the face ratio for female faces. In contrast, all four variables are highly inter-correlated in male faces, likely because these cues in male faces serve as “honest signals”.

## Introduction

Social interactions are better negotiated when we accurately gauge the behavioural propensities of others. The human face provides a good basis for such judgements. Facial expressions, for example, readily convey a person's emotional status and behavioural intentions [1], [2]. The ability to perceive facial expressions is adaptive in that it can facilitate the appropriate approach or avoidance behaviour [3]. There also is evidence that accurate perception of traits is possible from photographs of emotionally neutral faces: Significant correlations were found between observers' perceptions of and actual scores for “cheating” behaviour (in a Prisoner's Dilemma game [4]), for men's interest in infants [5], for men's strength [6], trustworthiness [7], history of violence [8], and aggressiveness [9]. A facial metric that may be involved in such judgements, particularly judgements of aggression, is the facial width-to-height ratio (face ratio) [10]).

The face ratio, first reported by Weston, Friday, and Liò [11], is a sexually dimorphic facial characteristic that, unlike other sexual dimorphisms in the face, is independent of body size. The sexual dimorphism appears at puberty when the growth trajectories of male and female skulls diverge for bizygomatic width, but not height. This divergence leads to a greater width-to-height ratio in male faces relative to female faces [11]. Changes in skull growth are linked to testosterone concentrations during puberty [12]. We previously found that the face ratio in men was correlated positively with their aggression during the Point Subtraction Aggression Paradigm (PSAP [10]). The PSAP is a well validated behavioural measure of aggression [13], guised as an online competitive computer game in which participants are made to believe they are playing against another person. We also found the face ratio in men was associated positively with aggression (as measured by the number of penalty minutes per game) in varsity and elite ice hockey [10]. Other researchers have reported the face ratio of men predicted behaviour aimed at exploiting the trust of others for personal gain [7], cheating behaviour, and the explicit use of deception [14]. Therefore, the face ratio may serve as an accurate signal of aggressive and trustworthy behaviours, the perception of which are highly negatively related (r = −.90 [9]).

To determine if the face ratio is used by observers for judgements of aggression, we conducted a series of studies in which observers were asked to judge propensity for aggression from pictures of male faces whose aggressive behaviour and face ratio previously were quantified [10]. The ratings of aggression were found to be correlated with both the participant's face ratio and the participant's aggression during the PSAP. Additionally, the association between the face ratio and estimates of aggression persisted even when the same stimuli were displayed for as little as 39 ms [9], blurred (to prevent judgements based on individual facial features [15]) or cropped (to maintain the face ratio yet remove influence of forehead, chin, and ears [15]). In another study, adult and 8-year-old White (from Canada) and Asian (from China) observers judged aggression in same- and other- race male faces (White and Asian faces [16]). For all observers, judgements of aggression irrespective of the race of the face were associated with the face ratio. Therefore, the face ratio seems to be an important signal that observers use for judging aggression. To date, we have not investigated the relationship between the face ratio and judgements of aggression in female faces.

Because the face ratio is sexually dimorphic and because the relationship between the face ratio and actual behaviour was found for men and not for women [7], [10], [14], the face ratio may not be a cue used by observers for judging aggression in female faces. In fact, characteristics of the female face, in general, may be less useful for predicting actual behaviour: Sell and colleagues [6] found that estimates of strength from photographs of the female face were less accurate than those from photographs of the male face. Judgements of dominance in chimpanzees, a species for which there is a similar sexually dimorphic facial ratio to that in humans [11], were more accurate for male than for female chimpanzee faces [17]. On the other hand, researchers have shown that irrespective of the sex of the face, observers use similar cues (e.g., facial masculinity) when making judgements about traits related to aggression (e.g., dominance [18]–[21]). It is thus worthwhile to investigate the influence of the face ratio on observers' perceptions of aggression in female faces.

Here, two studies were conducted to explore the relationships between the face ratio, judgements of aggression, masculinity/femininity, and attractiveness. In Study 1, we investigated whether or not observers' judgements of aggression were associated with the face ratio in female faces, as was found previously for male faces. We also determined if the face ratio was associated with judgements of aggression when the influence of judgements of masculinity (in male faces) or femininity (in female faces) was controlled statistically. In Study 2, we investigated the relationship between judgements of masculinity and of aggression in female faces, and again determined whether or not the face ratio was associated with judgements of aggression when the influence of masculinity was controlled statistically.

A third study was added to investigate whether differences in the relationships for female faces and male faces observed in Study 1 and Study 2 between ratings of masculinity and femininity and aggression are because of the use of a stereotypically male trait. In Study 3, we investigated whether judgements of nurturing, a stereotypically female trait, were more strongly associated with judgements of femininity in female faces, compared to in male faces. Although our previous research found no evidence that the correlations between the face ratio and ratings of aggression differed for men and women observers [9], [16], we included sex of observer in initial statistical analyses because of the possibility that men are more accurate than women when judging female faces (e.g., [6]).

## Materials and Methods

### Ethics Statement

The studies received ethics clearance by the Brock University Research Ethics Board, and all participants provided written, informed consent.

### Participants

Participants were recruited through an online undergraduate research pool and received a $5 honorarium or a course-related credit. Study 1: 40 participants (20 male, mean age = 20.63, SD age = 2.71, age range: 18–28 years , 75% White, 25% other); Study 2: 20 participants (10 male, mean age = 22.30, SD age = 3.21, age range: 18–32 years, 80% White, 20% other); and Study 3, 40 participants (20 male, mean age = 19.95, SD age = 1.55, age range: 18–25 years, 75% White, 25% other).

### Stimuli

**Figure 1:**
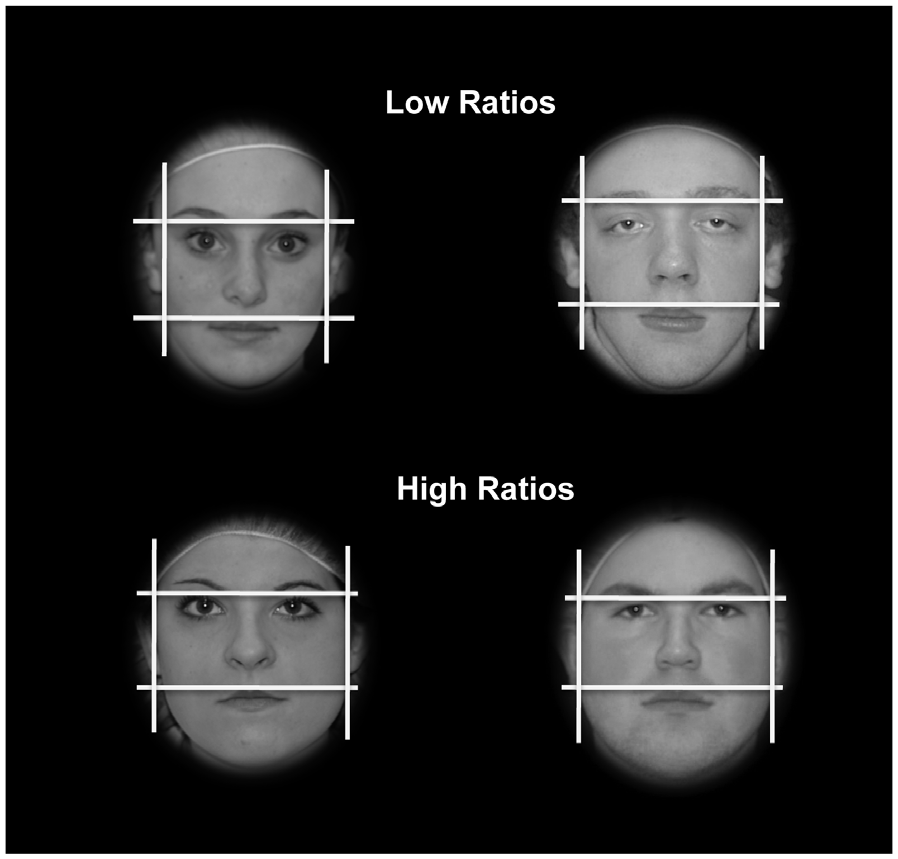
Example of female and male stimuli used in Studies 1, 2 and 3. The faces differ in width-to-height ratio (face ratio). The lines drawn on the faces were not shown to observers and are included here to illustrate the landmarks used to measure the face ratio.

Photographs were selected from a set of 37 male and 51 female participants (mean age = 18.98 years, SD age = 1.15, 82% White, 18% other) for whom the relationship between aggressive behaviour and facial width-to-height ratio previously was examined (r = .38 for men, r = −.05 for women; see [10]). Aggressive behaviour was measured using the Point Subtraction Aggression Paradigm, a well validated behavioural measure of aggression [13]. Participants were photographed with a Nikon D50 digital camera while posing in a neutral facial expression and wearing hair nets to conceal hair style. Photos were standardized with a hairline to chin distance of 400 pixels, 8-bit greyscale, and were elliptically cropped (with a black background) to ensure only the face of the stimuli was visible. Face ratio was calculated using NIH ImageJ software and the Weston et al. [11] landmarks: We divided the distance between the left and right zygion (bizygomatic width) by the distance between the mid-brow and upper lip. The number of male faces was reduced to 24 (mean age = 19.08 years, SD age = 1.41 years, mean face ratio = .83, SD face ratio = 0.138) by excluding self-identified non-Whites and faces with facial hair to avoid observer judgements based on stereotypes, and was the set of 24 male faces used in other studies investigating the relationship between the face ratio and ratings of aggressive behaviour [9], [15], [16]. The set of female faces was reduced to 31 (mean age = 18.87, SD age = 1.09, mean face ratio = 1.79, SD face ratio = 0.097) by excluding self-identified non-Whites and faces that were not posed in a neutral expression. With the smaller set of faces the higher face ratio of male than female faces approached statistical significance (t_53_ = 1.60, *p* = 0.06) (the difference was significant in the larger original sample of 88 faces). The correlations between the face ratio and actual aggression in the reduced sample of female (r = .17) and male faces (r = .31) were of similar magnitude as with the larger sample of faces in which the association was significant for male faces only, but the correlation was no longer statistically significant in the reduced sample of male faces. See Figure 1 for examples of low and high face ratio faces. Photos of females with visible jewellery (e.g., earrings) were modified using Adobe Photoshop to erase the visible jewellery to avoid observer bias in judgements.

### Statistics

Analysis of variance (ANOVA) was used to investigate for any effect of sex of observer or interaction of sex of observer and sex of face in the correlations between face ratings and face ratio before averaging ratings across all participants. The relationship between the face ratio and ratings were investigated using Pearson product moment correlations, Fisher z transformations of the correlations for use in ANOVA, chi square test, and multiple linear regression. An alpha of p<0.05, two-tailed, was used to determine statistical significance. Post hoc analysis, where applicable, consisted of Bonferroni corrected t-tests. Cronbach's alpha was calculated to examine the consistency of the ratings across individual participants. In tables of bivariate correlations, those that are significant both before and after Bonferroni correction are shown because the uncorrected correlations were consistent with findings from omnibus analyses (multiple linear regression) and consistent across studies. Thus, while the use of highly conservative Bonferroni corrections may have decreased Type I error, Type II error may have increased.

### Study 1: Are judgements of aggression made by observers correlated with the face ratio in female faces as they are in male faces?

#### Purpose

The primary purpose of this study was to investigate whether observers' ratings of aggressiveness were associated with the face ratio in female faces. In male faces, we previously found that ratings of aggressiveness were highly correlated with dominance (r = .92), masculinity (r = .86), and with attractiveness (r = −.57), but only ratings of aggressiveness and dominance were correlated with the face ratio (r = .59 and .54, respectively [9]). We thus investigated whether the same pattern of correlations would be found in female faces. The inclusion of judgements of attractiveness also enabled us to investigate the relationship between masculinity and attractiveness in male faces, given this relationship is not well understood: Some researchers have found a negative association, whereas other researchers have found a positive association between these ratings (see [22]).

#### Procedure

Images of the face stimuli were approximately 17 cm wide by 20 cm high (or 15.2×12.9 visual degrees when viewed from 75 cm) and presented using E-Prime software and a 17 inch Dell laptop monitor. Before the presentation of any stimuli, participants were told how aggressive behaviour had been assessed and prior to making any judgements participants viewed each face for 1000 ms to be familiarized. Half of the participants of each sex rated female faces and the other half rated male faces. For each set of female and male faces, the order of faces was randomized across participants. After familiarization, each participant rated the faces on three different characteristics, and all participants did so in the same order: aggression, masculinity (male faces) or femininity (female faces), and attractiveness.

During the rating tasks, each face was presented for 2000 ms after which a question appeared. Once the observer made a response using a Dell Laptop standard keyboard (observers were given unlimited time to make a response), the next photo was displayed. This process continued until all of the photos were rated on all three questions. The specific questions were: “How aggressive would this person be if provoked?”, “How masculine (or feminine, for the female faces) does this person look?”, and “How attractive does this person look?”. Ratings were made using a 7 point Likert scale (1 = *not at all*, 7 = *very much so*). Thus, each face was presented four times, once for familiarization and once for each of the three ratings.

### Study 2: Are ratings of masculinity associated with ratings of aggressiveness in female faces?

#### Purpose

Here, we investigated whether ratings of masculinity and of aggression in female faces are correlated, and whether the ratings are correlated with the face ratio.

#### Procedure

Only the set of female faces was used in Study 2. The stimulus faces were presented as in Study 1, including the pre-exposure to the faces, except that instead of rating femininity in the face, participants rated the masculinity of the female faces.

### Study 3: Are ratings of nurturing associated with ratings of femininity in female and male faces?

#### Procedure

The female and male faces were presented as in Study 1, including a pre-exposure to the faces before rating the faces. Participants were asked to rate “How nurturing is this person,” which appeared on a black background with a 7 point Likert scale (1 = *not at all nurturing*, 7 = *very nurturing*). Participants were provided a definition of nurturing before making ratings: “Nurturing has been defined as the process of caring for and encouraging the growth or development of someone (e.g., pet, friend) or something (e.g., plant)”. This definition of nurturing was chosen to avoid explicit mention of caring for children, to reduce the explicit sex-specificity of the rating [30]. Participants then rated the femininity and the attractiveness of the faces.

## Results

### Study 1 Results

#### Descriptive statistics for the ratings of female and male faces

The estimates of aggression, femininity, masculinity, and attractiveness were highly consistent across the 20 individual observers (all *Cronbach's alphas* >.90). The average ratings of aggression, masculinity/femininity, and attractiveness for the 20 men and 20 women observers were calculated. For ratings of aggression the interaction of Sex of Observer and Sex of Face was significant, F_1,53_ = 7.058, *p*<0.01 (see Table 1). No post hoc Bonferonni corrected t-test was significant (all p>0.0125).

**Table 1:** Descriptive statistics for ratings of female (n = 31) and male (n = 24) faces in Study 1.

	Aggression		Femininity	Masculinity	Attractiveness*	
	Mean (*S.D.*)		Mean (*S.D.*)	Mean (*S.D.*)	Mean (*S.D.*)	
Observers	Females	Males	Females	Males	Females	Males
Women (n = 10)	3.94	4.18	4.28	4.55	3.65	3.10
	(1.01)	(0.79)	(1.10)	(.99)	(1.17)	(0.98)
Men (n = 10)	4.15	4.00	4.05	4.19	3.38	2.86
	(0.80)	(0.96)	(1.01)	(.88)	(1.02)	(0.76)
**Total** (n = 20)	4.05	4.09	4.16	4.37^a^	3.52^b^	2.98^b^
	(0.87)	(0.83)	(1.01)	(.88)	(1.05)	(0.85)

Matched letters indicate significantly different ratings between sex of face stimuli, p<0.05;

*Main effect of sex of observer for a rating type, p<0.05.

The ratings of femininity in female faces were lower than ratings of masculinity in male faces, F_1,53_ = 11.598, *p* = 0.001, irrespective of the sex of the observer. Female faces were rated as more attractive than were male faces F_1,53_ = 4.175, *p* = 0.05, and women observers rated both sets of faces higher on attractiveness than did men observers, F_1,53_ = 11.07, *p* = 0.001.

#### Relationship between ratings of aggression and the face ratio: analysis of correlations of individual observers

**Figure 2:**
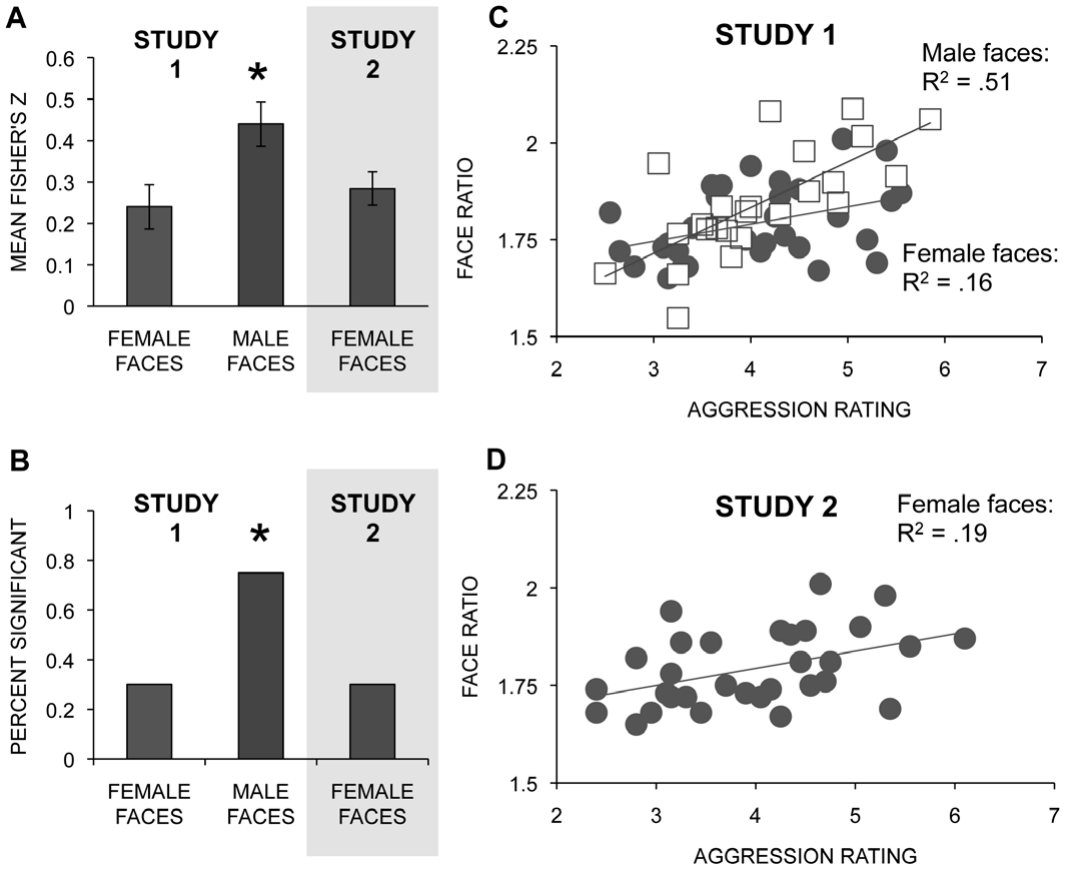
Bar graphs and scatterplots showing the relationship between the face ratio judgements of aggression. **A** The mean Fisher's Z correlations between observers' judgements of aggression and the face ratio in Study 1, for male (n = 24) and female faces (n = 31), and, in Study 2 (shaded area), for female faces (n = 31). Error bars represent the standard error. * male faces > female faces, p<0.05. **B** The percent of observers whose judgements of aggression were correlated significantly with the face ratios of male (n = 24) and female faces (n = 31) in Study 1, and female faces (n = 31) in Study 2 (shaded area). **C** Scatterplot of the face ratio and judgements of aggression in male (n = 24) and female faces (n = 31) in Study 1. **D** Scatterplot of the face ratio and judgements of aggression in female faces (n = 31) in Study 2.

To determine whether any relationship between ratings of aggression and the face ratio was stronger for male or for female faces, Fisher z correlations between the face ratio and the estimates of aggression were calculated for each individual observer. A Sex of Face X Sex of Observer ANOVA found that correlations were higher for male faces than for female faces, F_1,36_ = 7.43, *p* = 0.01 (see Figure 2-A), and did not differ for men and women observers.

Based on Pearson correlations, for female faces, 6 of 20 correlations for observers were significant (r>.36, two-tailed), and for male faces, 15 of 20 correlations for observers were significant (r>.40, two tailed). The proportion of significant correlations was higher for male faces than it was for female faces (χ^2^ = 3.86, p<0.05) (see Figure 2-B).

#### Relationship between mean ratings of aggression across observers and the face ratio

Here we used linear regression to test whether the face ratio was a better predictor for male faces than for females faces of the mean estimates of aggression across observers. Sex of Face and the Face Ratio were entered on the first step of the regression, and the interaction of Sex of Face and Face Ratio was entered on the second step. The first step of the model was significant (adjusted R^2^ = .27, F_2,52_ = 11.26, p<0.0001), with the Face ratio the only significant predictor (t = 4.73, p<0.0001). The addition of the interaction term did not significantly increase predictive power (R^2^ change = .002, F change = .17, p = .68). The correlation between the face ratio and ratings of aggression was r = .71 (p<0.0001) for male faces and r = .40 (p = 0.03) for female faces (see Figure 2-C). Thus, in contrast to the finding of higher correlations for male than for female faces using the correlations between individual ratings of aggression and the face ratio, the higher association between ratings of aggression and the face ratio for male faces than for female faces is not significant when averaged across observers.

#### Correlations among ratings

For female faces, ratings of aggression were not associated with ratings of attractiveness or of femininity, which were highly correlated (r = .91) and no rating other than ratings of aggression were associated with the face ratio (see Table 2). For male faces, the only correlations that were not significant were between ratings of attractiveness and the other variables (see Table 2).

**Table 2:** Pearson product moment correlations between the face ratio and face ratings in Study 1.

	Ratings of Female faces (n = 31)			Ratings of Male faces (n = 24)		
	Aggression	Feminine	Attractive	Aggression	Masculine	Attractive
Face Ratio	**.40**	.05	−.01	**.71**	**.46**	−.24
	**p = 0.03**	p = 0.80	p = 0.97	**p<0.001** *	**p = 0.03**	p = 0.25
Aggression		−.04	.01		**.83**	−.39
ratings		p = 0.83	p = 0.96		**p<0.001** *	p = 0.06
Fem/Masc			**.96**			−.17
ratings			**p<0.001** *			p = 0.44

Correlations in boldface are significant, *p*<.05, two-tailed.

*significant after Bonferonni correction (p<0.004).

#### Is the face ratio a basis for the ratings of aggression?

**Figure 3:**
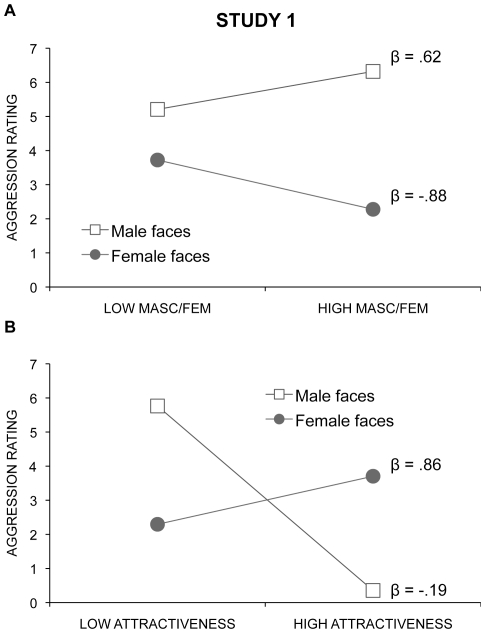
Interaction plots between sex of face and masculinity, and sex of face and attractiveness in the linear regression predicting aggression ratings. **A** The interaction between the sex of the face and ratings of masculinity (in males) or femininity (in females) predicting judgements of aggression when controlling for ratings of attractiveness and the face ratio. **B** The interaction between sex of the face and ratings of attractiveness when controlling for the face ratio and ratings of masculinity. βs are the standardized regression coefficients, representing the unique influence of each predictor when controlling for the other variables that were entered into the model. High and low scores for the plots were calculated using scores 1 SD above and 1 SD below the means.

**Figure 4:**
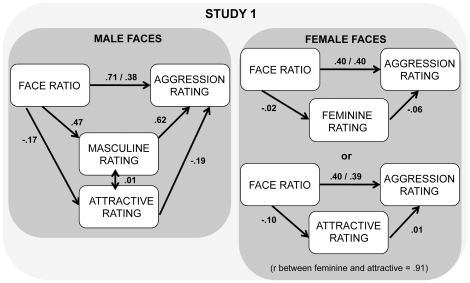
Face ratio accounted for unique variability in judgements of aggression over and above other predictors. Mediation models were used to determine if the face ratio remained a significant predictor of judgements of aggression in male (n = 24) and female faces (n = 31) when controlling for ratings of masculinity and attractiveness. The numbers shown are standardized regression coefficients, (β weights). In the mediation model used for male faces, face ratio was entered on the first step and ratings of masculinity and attractiveness were entered on the second step. The first standardized regression coefficient between face ratio and judgements of aggression is that when the face ratio alone is used as a predictor of judgements of aggression. The second standardized regression coefficient is that when face ratio and ratings of masculinity and attractiveness are entered on the same step as predictors. For female faces, because of the high redundancy between ratings of femininity and of attractiveness, two mediation models were used to examine the unique effect of the face ratio in predicting judgements of aggression first, over and above ratings of femininity and, second, over an above ratings of attractiveness. The first standardized regression coefficient between face ratio and judgements of aggression is that when the face ratio alone is used as a predictor of judgements of aggression. The second standardized regression coefficient is when the face ratio and ratings of femininity, or of attractiveness, are entered on the same step as predictors.

Based on the correlations, for male faces, the face ratio may be associated with aggression simply because it is correlated with masculinity. Because judgements of masculinity involve many cues in the face other than the face ratio (e.g., eye-mouth-eye angle [23]; fluctuating asymmetry [24]; jaw width, or width of face at mouthline [25]; eye size, lower face height/face height, cheekbone prominence, face width/lower face height, mean eyebrow height [26]), we used linear regression to test whether the face ratio continues to be associated significantly with ratings of aggression when ratings of masculinity (for male faces) or femininity (for female faces) and ratings of attractiveness are added to the model. The face ratio remained a significant predictor (beta value = .56, t = 3.219, p = 0.002) with the addition of the additional predictors and their interactions. Because the interaction of sex of face and ratings of masculinity/femininity and the interaction of sex of face and ratings of attractiveness were both significant (p<0.05) in the model (see Figure 3), we next calculated linear regressions for the sexes separately. For male faces, although adding masculinity and attractiveness ratings as predictors reduced the association between the face ratios and ratings of aggression (see Figure 4), the face ratio remained a significant predictor. Both masculinity (p<0.0001) and attractiveness ratings (p = 0.04) also were significant predictors. For female faces, because of the high correlation between femininity ratings and attractiveness ratings (r = .91), to avoid collinearity effects, separate regressions were performed using each rating alone on the second step. The addition of neither one resulted in a significant increase in predictive power (F change = .116 and .006, respectively, both p>.70), and neither were significant predictors of aggression ratings for female faces (p = .74 and p = .94) (see Figure 4).

#### Discussion of Study 1

Ratings of both male and female faces were highly consistent across observers. The face ratio was associated with ratings of aggressiveness in female faces, but not to the same extent as in male faces. The correlations between the face ratio and estimates of aggression of observers were smaller for female faces than for males faces, and fewer of the correlations of individual observers rating female faces were significant compared to observers rating male faces. Averaging the rating of aggression across observers, however, attenuated the sex difference in the relationship between estimates of aggression and the face ratio. We previously reported a relationship between actual aggressive behaviour and the face ratio in men and not in women [10]. Thus, the face ratio may not be an “honest signal” in women, and the association between the face ratio and ratings of aggression may reflect a generalization of a cue that may be meaningful in male faces to female faces.

The results provide additional evidence that the face ratio is indeed a basis for the estimates of aggression in faces. Although ratings of aggression were highly associated with ratings of masculinity in male faces, which was also associated with the face ratio, regression analyses indicated that the face ratio remained a significant predictor of ratings of aggression when controlling for ratings of masculinity in male faces, ratings of femininity in female faces, and ratings of attractiveness in either sex of face. The face ratio is only one of many cues of masculinity in the face. That the face ratio remained a significant predictor when controlling for the effects of other cues of masculinity is consistent with the finding of Weston and colleagues that the face ratio is independent of other sexual dimorphisms in the face [11].

We had expected that femininity would be negatively associated with aggression in female faces on the assumption that ratings of femininity are inversely related to masculinity. The lack of a relationship between ratings of femininity and of aggression in female faces may be because aggression is viewed as a masculine characteristic [27] and/or because ratings of femininity may be independent of ratings of masculinity, in which case correlations between judgements of masculinity and of aggressiveness may instead be found for female faces. We investigate this possibility in Study 2.

Consistent with other studies (e.g., [28], [29]), ratings of masculinity and attractiveness for male faces were not as highly correlated as were ratings of femininity and attractiveness in female faces. In male faces, the association was negative and non-significant, whereas the association in women was positive and significant. The relationships between masculinity/femininity and attractiveness also were tested in Studies 2 and 3 and are discussed further in the Discussion.

### Study 2 Results

#### Descriptive statistics

The estimates of aggression, masculinity, and attractiveness were highly consistent across the 20 individual observers (all *Cronbach's alphas* >.90). Men observers rated the female faces as less aggressive than did women observers (mean = 3.81 vs 4.12, t_30_ = 2.59, *p* = 0.015), but ratings of attractiveness (mean = 3.1 vs 3.1, *p* = 0.80) and of masculinity (mean = 3.8 vs 3.7, *p* = 0.30) did not differ between groups.

#### Relationship between ratings of aggression and the face ratio

Fisher z correlations between the face ratio and the estimates of aggression for females for each individual observer were calculated. The correlations did not differ based on the sex of the observer (Women Observers mean = .34, SD = .15; Men Observers mean = .23, SD = .19; t_18_ = 1.48 *p* = 0.16), and 6 of the 20 individual correlations were statistically significant (r>.36, p<0.05, two-tailed). The mean correlation for observers of female faces in Study 2 was smaller than that for observers of male faces in Study 1 (t_38_ = 2.40, p = 0.02) (see Figure 2-A) and did not differ from that for observers of female faces in Study 1 (t_38_ = 1.02, p = 0.32).

Mean ratings of aggression for female faces across observers in Study 2 and the face ratio was significant (r = .44 *p* = 0.01) (see Figure 2-D).

#### Are ratings of masculinity related to ratings of aggression in female faces?

**Figure 5:**
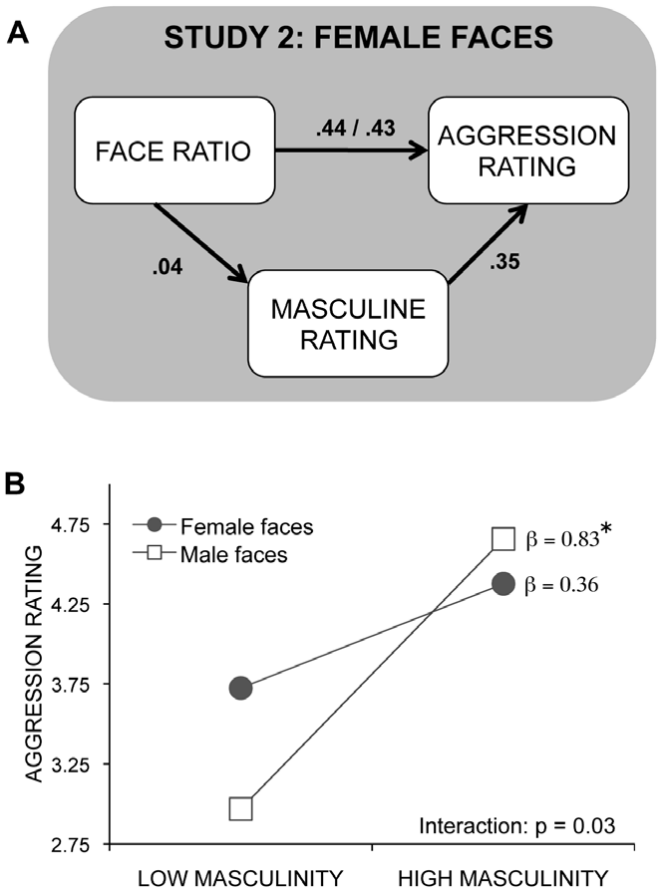
Mediation model and interaction plot with ratings of masculinity predicting judgements of aggression. **A** A mediation model was used to determine if the face ratio remained a significant predictor of judgements of aggression in female faces (n = 31) in Study 2 when controlling for ratings of masculinity. The numbers shown are standardized regression coefficients (β weights). The first standardized regression coefficient between face ratio and judgements of aggression is that when the face ratio alone is used as a predictor of judgements of aggression. The second standardized regression coefficient is that when face ratio and ratings of masculinity are entered on the same step as predictors. **B** Plot of the interaction between ratings of masculinity by sex of the face in predicting judgements of aggression. Low and high values represent scores 1 SD below and 1 SD above the mean.

As in Study 1, we used linear regression to test whether the face ratio continues to be a significant predictor of ratings of aggression when ratings of masculinity are added to the model. The addition of masculinity ratings to the model was significant (R^2^ change = .15, F change = 4.92, *p* = 0.035), but the face ratio remained a significant (p = 0.01) predictor of ratings of aggression in female faces (see Figure 5-A). The addition of attractiveness ratings instead of masculinity (not added together because of high collinearity) did not improve the prediction of aggressiveness ratings (R^2^ change = .000, F change = 0.008, *p* = 0.93).

We used linear regression to test whether the relationship between masculinity and aggression in female faces in Study 2 was as strong as it was for male faces in Study 1. Sex of face and masculinity ratings were entered on the first step and their interaction was entered on the second step. Both models were significant, the addition of the interaction term increased the prediction of aggression ratings (adjusted R^2^ = .30, R^2^ change = .065, F change = 4.97, p = .03), and the beta weight for male faces was greater than that for female faces (p = 0.005) (see Figure 5-B).

#### Are associations with ratings of masculinity comparable to those obtained in Study 1 with ratings of femininity?

Within Study 2, ratings of masculinity were correlated positively with ratings of aggressiveness (r = .36, p = 0.045) and negatively with ratings of attractiveness (r = −.88, p<0.0001) (see Table 3). Ratings of masculinity of female faces in Study 2 were not associated with ratings of aggressiveness of female faces in Study 1 (r = .25), but masculinity in Study 2 was highly associated with femininity in Study 1 (r = −.93, p<0.0001).

**Table 3:** Pearson product moment correlations between the face ratio and ratings of female faces (n = 31) in Study 2.

	Aggression	Feminine	Attractive
	ratings	ratings	ratings
Face Ratio	**.44**	.04	.06
	**p = 0.01**	p = 0.85	p = 0.75
Aggression		**.36**	.01
ratings		**p = 0.05**	p = 0.95
Fem/Masc			**.90**
ratings			**p<0.001** *

Correlations in boldface are significant, *p*<.05, two-tailed.

*significant after Bonferonni correction (p<0.008).

#### Discussion of Study 2

The face ratio was associated with ratings of aggression in female faces, although the correlations were lower than were obtained previously for male faces. Thus, the results are consistent with those of Study 1. There was a modest association between ratings of aggression and of masculinity, although none had been found for ratings of aggression and of femininity in Study 1, even though the ratings of masculinity in female faces were the inverse of ratings of femininity in Study 1. The lower association between ratings of masculinity and of aggression in female faces in Study 2 compared to in male faces in Study 1 may be because both masculinity and aggression are stereotypically male characteristics that best fit judgements of male faces. We thus investigated in Study 3 how ratings of femininity are associated with ratings of nurturing, a stereotypically female characteristic, in the female and male faces and the associations among ratings across the three studies. These results would allow us to test: (1) whether ratings of masculinity/femininity are relevant in female faces when judging a stereotypically female trait, and thus the sex difference in the relevance of masculinity/femininity in faces is specific to judgements of aggression, and/or (2) whether judgements of masculinity/femininity in faces is relevant only for the sex of face for which the trait is stereotypic. If the latter is true, we would predict that judgements of masculinity/femininity are associated with judgements of nurturing for female faces and not for male faces.

### Study 3 Results

#### Descriptive statistics

The ratings of nurturing did not differ for female and male faces, and did not differ for women and men observers, and there was no interaction of the two factors (see Table 4). Female faces were rated as more feminine (F_1,106_ = 33.64, *p*<0.0001) and as more attractive (F_1,106_ = 9.64, *p* = 0.002) than were male faces, and women observers gave higher ratings of attractiveness than did men observers (F_1,106_ = 3.82, *p* = 0.05).

**Table 4:** Descriptive statistics for ratings of female (n = 31) and male (n = 24) faces in Study 3.

	Nurturing		Femininity		Attractiveness*	
	Mean (*S.D.*)		Mean (*S.D.*)		Mean (*S.D.*)	
Observers	Females	Males	Females	Males	Females	Males
Women (n = 10/	4.15	3.96	4.44	3.03	4.07	3.08
sex of face)	(0.73)	(0.87)	(1.12)	(0.89)	(1.07)	(1.05)
Men (n = 10/	4.10	3.80	4.03	3.13	3.31	3.08
sex of face)	(0.90)	(0.77)	(1.00)	(1.10)	(1.04)	(0.94)
**Total** (n = 20/	4.12	3.88	4.23^a^	3.08^a^	3.69^b^	3.08^b^
sex of face)	(0.79)	(0.80)	(1.03)	(0.95)	(1.04)	(0.97)

Matched letters indicate significantly different ratings between sex of face stimuli, p<0.05;

*Main effect of sex of observer for a rating type, p<0.05.

#### Are ratings of nurturing in female and male faces associated with other ratings?

Nurturing ratings were associated with femininity and with attractiveness ratings of female faces (all rs>.52, *p*<0.05) and male faces (all rs>.78, *p*<0.05). Femininity and attractiveness were highly correlated in female faces and in male faces (all rs>.57, *p*<0.05) (see Table 5).

**Table 5:** Pearson product moment correlations between ratings of female faces (n = 31) and male faces (n = 24) in Study 3.

	Female faces		Male faces	
	Feminine	Attractive	Feminine	Attractive
Nurturing	**.67**	**.59**	**.83**	**.84**
	**p<0.001**	**p<0.001**	**p<0.001**	**p<0.001**
Feminine		**.96**		**.74**
		**p<0.001**		**p<0.001**

All correlations were significant after Bonferonni correction (p<0.008).

#### Patterns of correlations across studies: Do constructs of aggression and nurturing map on to constructs of masculinity and femininity?

For both male and female faces, ratings of masculinity and of femininity were highly negatively correlated and ratings of aggression and of nurturing were highly negatively correlated. For both male faces and female faces, nurturing was associated negatively with masculinity and positively with femininity. What differed for male and female faces was the association of aggression to masculinity/femininity. For male faces, aggression was associated positively with masculinity and negatively with femininity (see Table 6). For female faces, neither of the two correlations between femininity and aggression were significant and only one of the two correlations between masculinity and aggression were significant (see Table 6).

**Table 6:** Pearson product moment correlations between ratings of aggression, masculinity, nurturing, and femininity and with the face ratio and actual aggression across Study 1 (S1), 2 (S2), and 3 (S3).

	Female Faces (n = 31)					
	AggressiveS1	Aggressive S2	Masculine S2	Nurturing S3	Feminine S1	Feminine S3
Aggressive S2	**.88**					
	**p<0.001** *					
Masculine S2	.25	**.36**				
	p = 0.18	**p = 0.05**				
Nurturing S3	**−.6**	**−.69**	**−.76**			
	**p<0.001** *	**p<0.001** *	**p<0.001** *			
Feminine S1	−.04	−.18	**−.93**	**.64**		
	p = 0.82	p = 0.32	**p<0.001** *	**p<0.001** *		
Feminine S3	−.04	−.16	**−.94**	**.67**	**.95**	
	p = 0.82	p = 0.39	**p<0.001** *	**p<0.001** *	**p<0.001** *	
Face Ratio	**.40**	**.44**	.04	−.14	.05	
	**p = 0.03**	**p = 0.01**	p = 0.82	p = 0.44	p = 0.80	
Actual	−.04	−.06	−.07	.08	.07	.06
aggression*	p = 0.82	p = 0.75	p = 0.73	p = 0.68	p = 0.73	p = 0.75

	Male Faces (n = 24)					
	Aggressive S1		Masculine S1	Nurturing S3		Feminine S3
Masculinity S1	**.83**					
	**p<0.001** *					
Nurturing S3	**−.69**		**−.55**			
	**p<0.001** *		**p = 0.005**			
Femininity S3	**−.79**		**−.83**	**.83**		
	**p<0.001** *		**p<0.001** *	**p<0.001** *		
Face Ratio	**.71**		**.46**	−.33		−.36
	**p<0.001** *		**p = 0.03**	p = 0.11		p = 0.08
Actual	**.41**		.39	−.36		**−.45**
Aggression#	**p = 0.05**		p = 0.06	p = 0.08		**p = 0.03**

# Obtained in a previous study [10]. Correlations in boldface are significant, *p*<.05.

*significant after Bonferonni correction (p<0.002).

Table 6 also shows the correlations between the ratings of the faces and actual aggression (PSAP scores previously obtained). For female faces, no correlation accounts for more than 1% of the variance in actual aggression, for male faces, each correlation accounts for over 10% of the variance (range of 12% to 16%) in actual aggression.

## Discussion

The main findings of the studies are that, first, we extend our previous reports that the facial width-to-height ratio (face ratio) is predictive of observers' judgements of aggressive potential in male faces [9], [15], [16] to female faces. Secondly, we provide new evidence to support our previous assertion that the face ratio is a critical cue in judgements of aggression. And thirdly, we provide evidence that for observers, masculinity and femininity are inversely related and not orthogonal for both male and female faces. Nevertheless, the construct of aggression is not related to femininity and modestly correlated with masculinity for female faces, but is strongly related to constructs of masculinity/femininity for male faces. Further, the construct of nurturing is related to constructs of masculinity/femininity for both male and female faces.

### The association between the face cues and judgements of aggression is stronger for male faces than for female faces

When using the correlations between aggression ratings and face ratios for individual observers, those for male faces were higher than those for female faces. Further, the number of significant correlations between the face ratio and ratings of aggression of individual observers was higher for male faces than for female faces (75% vs 30% of correlations). When the ratings were averaged across observers, the face ratio accounted for more of the variance in ratings of aggression in male faces (50%) than in female faces (16%), but the difference was not statistically significant.

There are a number of possibilities as to why the relationship is stronger for male faces than for female faces. One possibility is the face ratio is not predictive of actual behaviour in women as it is in men. For example, the face ratio in women was not associated with aggression in the laboratory (measured using the PSAP) whereas the face ratio in men was associated with aggression both inside and outside of the laboratory (measured as penalty minutes per game [10]). In a separate study [7], men with larger face ratios exploited the trust of others for personal gain more frequently than did men with smaller face ratios, but there was no such relationship for women. A recent study also found that the face ratio was associated with deceptive, unethical behaviour, but only in men and not in women [14]. Thus, there is no evidence to suggest that the face ratio specifically, or the face in general, is an “honest signal” of aggressive potential in women's faces, which may be a reason why the correlations between estimates of aggression and the face ratio were smaller for women than for men.

Some researchers have suggested that misjudging the aggressive potential of women may be less costly than would be misjudging the aggressive potential of men [6]. Because men are more physically aggressive than are women (e.g., in adulthood [31], [32]), observers may not be as cognitively specialized for assessing aggression in women as they are in men [6]. From an ecological perspective, the perception of emotions likely serves an adaptive function [1]. The perception of angry expressions is adaptive in that they warn others of one's aggressive intentions and thus should [1], [2], and do [3] provoke avoidance. Research on the perception of angry facial expressions supports the possibility of sex-specific perceptual abilities. The expression of anger, although universally understood [33], takes longer to be identified and is less accurately identified in female faces than in male faces [34]. Thus, not only are observers less accurate in their estimates of actual aggression, they are also slower and less capable of identifying cues related to aggression in females compared to in males.

We have argued that the face ratio may represent a more subtle signal of aggressive potential, and that a more overt signal, such as an angry facial expression, may serve to amplify this signal [15]. An angry facial expression involves the lowering of the brow and the raising of the upper lip [35], muscle movements that notably increase the face ratio [9]. Thus, the ability to make accurate estimates of aggression may be related, in part, to an overgeneralisation of emotional expressions [1], [36], [37], whereby individuals that have facial metrics that resemble a particular emotional state will be perceived as actually showing that emotion.

Our perceptual system may be so well adapted for assessing aggression in men that our perception of anger may be synonymous with our perception of masculinity. A face with a lowered brow ridge (which made the face look more angry) was interpreted as more masculine than was a face with a neutral expression [34], and when asked to imagine an angry face, observers were more likely to report visualizing a male [34]. Further, when shown a picture of an androgynous face with an angry expression, observers were more likely to perceive the stimuli as male than as female compared to when shown the androgynous face with a neutral expression [38].

Nevertheless, our findings suggest that judgements of masculinity/femininity may not be as relevant for estimating aggression in female faces as they are for male faces. Judgements of masculinity and of femininity were highly negatively correlated in our studies, to the extent that one could be the inverse of the other. In male faces, both masculinity (positively) and femininity (negatively) were associated with aggression, and both were associated with nurturing (each in the opposite direction as to aggression). In female faces, the association between masculinity and aggression was weaker than in male faces and femininity was not associated with aggression. Masculinity (negatively) and femininity (positively) were associated with nurturing in female faces. Thus, whereas judgements of nurturing were applied equally to male and female faces and their feminine/masculine appearance, judgements of aggression differed depending on the sex of the face being judged.

Previous studies in which observers were shown pairs of faces (one feminised and one masculinised version of each face) and asked to select which is most dominant (e.g., [18], [19], [21]) have found that observers selected the masculinised versions of both male and female faces significantly more than the feminised versions as being more dominant. Nevertheless, the effect sizes from these studies are smaller for female faces than for male faces [39]. That is, observers chose the masculinised face less frequently when selecting the most dominant from female face pairs than when selecting the most dominant from the male face pairs. Thus, our results are consistent with the literature in indicating that the association between perceptions of femininity/masculinity and judgements of aggression are weaker when judging aggression in female faces than when judging aggression in male faces.

Another possibility for the discrepancy in correlations between the face cues and aggression in male and female faces is that larger correlations would be found for female faces if observers were asked to judge a different type of aggression than reactive aggression. When asked to choose the most physically dominant between a pair of faces (i.e., someone who would likely win a fistfight against a same-sex opponent [40]), observers selected the masculinised female and male faces more frequently than the feminised female and male faces [21]. In contrast, when asked to choose the most socially dominant (i.e., someone who tells others what to do, is respected, influential, and a leader [41]) from a pair of female and male faces, observers selected the feminised versions of female faces but the masculinised versions of the male faces more frequently than the alternatives [21]. Thus, discrepancies between the cues used to judge female and male faces may also exist as a function of the type of aggression being judged.

### The face ratio is a critical cue in judgements of aggression

The face ratio remained a significant predictor of judgements of aggression, even when other cues in the face related to masculinity were controlled statistically. This finding strengthens our conclusion that the face ratio is a key basis for observers' judgements of aggression. Previously, we found that the face ratio continued to be associated with judgements of aggression when photographs were blurred reducing the ability to discriminate features or were cropped thereby removing other cues of masculinity (e.g., jaw line), but not when the faces were segmented thereby preserving all features but disrupting the face ratio [15]. We also showed that the face ratio was the only significant predictor of judgements of aggression when other cues in the face were included as predictors in a regression model [15].

We propose that the face ratio is an adaptation shaped by intra- and intersexual selection as a signal of aggressiveness and trustworthiness in male faces. The face ratio is distinct from other adaptations in the face. Other sexual dimorphisms in the face postulated to reflect selection pressure involve regions of the face that grow allometrically [11]. In contrast, the face ratio is a sexually dimorphic feature that is independent of selection pressure on body size and that develops at puberty coincident with the rise in testosterone in males [11]. In addition to growing evidence that it is an “honest signal” of aggressive and trustworthy behaviour in men and not in women (e.g., [7], [10], [14]), we recently showed that the face ratio is the basis for judgements of aggressiveness in men irrespective of the race of the face being rated [16]. The correlations between ratings of aggression and the face ratio of White observers in Canada and Asian observers in China, despite little exposure to the other race, were the same for Asian faces as for White faces. Further, we found that the judgements of aggression of Asian and White 8 year olds' also were associated with the face ratio irrespective of the race of the face being judged [16]. Thus, the cognitive mechanism that allows the detection and use of the face ratio appears to be broadly tuned and to function independently of experience. Further, we posit that the cognitive mechanism is specific to the detection of threat; there was no association between the face ratio and judgements of nurturing (or attractiveness), even though judgements of nurturing and aggression were highly negatively correlated.

### Femininity and nurturing, not aggressiveness, are attractive

Female and male faces that were perceived as more feminine were rated as more attractive. For female faces, our results are consistent with the literature: Femininity and attractiveness frequently are found to be associated positively (e.g., [28], [29] see [22] for meta-analysis). For male faces, our results are congruent with Perrett and colleagues [19] and Rhodes and colleagues [42] who found that feminised male composite faces were chosen as more attractive compared to masculinised male composite faces. Our study, however, finds a positive relationship between femininity and attractiveness in male faces using individual faces as stimuli (i.e., not using masculinised/feminised composite faces). Although secondary sex characteristics (e.g., masculine facial characteristics) may imply good health (immunocompetence; see [43]), they may also signal negative traits related to poor paternal investment potential [19]. Indeed, previous studies have found that masculine male faces, compared to feminine male faces, are rated as less committed and faithful to a long term relationship, less warm [44], and more antisocial [45], which is consistent with our finding of no relationship between the face ratio and attractiveness. Further, femininity was associated positively and masculinity was associated negatively with judgements of nurturing, and nurturing was associated with attractiveness whereas aggressiveness was not. Thus, our study is consistent with the literature and adds support to the idea that facial femininity/masculinity signals paternal investment potential with individuals perceived as being more feminine/less masculine perceived as more paternally investing [19], [44]. Further, our findings are consistent with studies showing that men with greater as opposed to less paternal investment potential (i.e., interest in infants) are more attractive (for long term relationships [5]).

### Conclusion

In summary, these studies indicate that the perception of aggression in female faces is different from that in male faces. The sex difference is not simply because aggression is a gendered construct; the relationships between masculinity, femininity, and nurturing were similar for male and female faces even though nurturing is arguably as gendered a construct as aggressiveness. The association between cues in the face and estimates of aggression is stronger for male than for female faces, likely because they serve as an “honest signal” in men, such as those found in other species [46]–[48].
